# The development of a novel Orthodontic Alignment Index and its use to evaluate the effect of residual overjet on the stability of the alignment of the maxillary anterior dentition

**DOI:** 10.1186/s40510-022-00444-1

**Published:** 2022-12-28

**Authors:** Ciarán P. Devine, Devaki Patel, Nikolaos Pandis, Padhraig S. Fleming

**Affiliations:** 1 Queen Mary University London, London, UK; 2 Cheam, Surrey, UK; 3 Department of Orthodontics and Dentofacial Orthopedics, Medical Faculty, Dental School, University of Bern, Bern, Switzerland; 4 Dublin Dental University Hospital, Trinity College Dublin, Ireland and Queen Mary University London, London, UK

**Keywords:** Overjet, Residual overjet, Relapse, Stability, Alignment

## Abstract

**Background:**

It is thought that achieving a normal overjet may help to stabilise the alignment of the maxillary anterior dentition. Little’s Irregularity Index is limited in assessing discrete post-orthodontic changes, fails to account for reciprocal rotations and is not sensitive to dental changes in three planes. A more holistic tool for the assessment of post-treatment change is therefore required.

**Aim:**

To compare the post-treatment stability of maxillary anterior dental alignment in subjects treated either to a Class I incisor relationship or an increased overjet (> 4 mm) following fixed appliance-based orthodontics using a novel measurement tool.

**Materials and methods:**

The Orthodontic Alignment Index (OAI) was developed and validated using a panel of 63 raters. The new index accounts for a range of weighted features including contact point displacement, spacing, reciprocal rotations, inclination, angulation and vertical discrepancy. A retrospective cohort study was undertaken at the Institute of Dentistry, Queen Mary University of London. Recruitment took place over a 4-year period. All participants had removable retainers in the maxillary arch only. The stability of maxillary anterior teeth was assessed using Little’s Irregularity Index (LII) and the OAI. Subjects were recruited at least 12 months following completion of dual-arch fixed appliance-based treatment.

**Results:**

Eighty-two participants were included with a positive correlation observed between LII and OAI at the 12-month post-treatment review with a 1-mm increase in LII associated with a 2-point increase in the OAI (*P* < 0.001). Limited relapse was observed in both groups: normal overjet group (OAI = 1.28; LII = 0.52); residual overjet group (OAI = 0.88; LII = 0.47). Median regression analysis failed to identify a significant association between an increased overjet at debond and the alignment of the maxillary anterior segment when assessed with OAI (*P* = 0.389) and LII (*P* = 0.577). Furthermore, age, gender, extraction protocols and retention regime were not predictive of post-treatment change.

**Conclusions:**

Using a novel index (OAI) and LII, there was limited post-treatment relapse in alignment of the maxillary anterior dentition over a 12-month period. Based on this retrospective evaluation, achieving a normal overjet at the end of treatment may have little bearing on the post-treatment stability of maxillary anterior alignment at 12 months.

## Introduction

A Class I incisor relationship is typically an objective of a comprehensive orthodontic treatment plan. There is a belief that this relationship enhances the stability of the maxillary labial segment post-orthodontic treatment. However, an increased and residual overjet may arise in certain situations. Specifically, in the presence of a moderate-to-severe Class II skeletal discrepancy, a Class I incisor relationship may not be achievable with orthodontics alone. A retrusive soft tissue pattern may also contra-indicate retraction of the maxillary labial segment for aesthetic reasons [1]; therefore, acceptance of a residual overjet may occasionally be preferable in order to balance occlusal and aesthetic dictates. Excessive overjet may also be an unplanned consequence of treatment, stemming from poor patient compliance, inappropriate planning or poor execution of a treatment plan.

There has been a trend towards the provision of short courses of orthodontic treatment with limited treatment objectives. This may entail suboptimal outcomes, such as the acceptance of a residual overjet [2]. It is postulated that a Class I incisor relationship with a normal overjet may have an additional stabilising effect on the alignment of the maxillary incisors; however, there are limited data to support this contention [2].

Little’s Irregularity Index (LII), Peer Assessment Rating (PAR) and the American Board of Orthodontics-Objective Grading System (ABO-OGS) are the most commonly used indices to assess the stability of the maxillary anterior segment. These indices are limited in assessing discrete post-orthodontic changes and relapse and are generally not sensitive to dental changes in three planes.

The aim of this study was to compare the post-treatment stability of maxillary anterior dental alignment in subjects treated to a Class I incisor relationship (2–4 mm) relative to subjects treated to an overjet in excess of 4 mm following fixed appliance-based orthodontic treatment.

## Materials and methods

A retrospective cohort study was carried out in the Orthodontic Department at the Institute of Dentistry, Barts and The London School of Medicine and Dentistry, Queen Mary University of London. Approval was obtained from the Barts NHS Health Trust Clinical Effectiveness Unit (ID 6274).

A convenience sample was recruited at least 12 months following the completion of dual-arch pre-adjusted fixed appliance-based treatment. Participants of all ages, skeletal and dental relationships having received removable retention (vacuum-formed retainers or Hawley retainers) in the maxillary arch were included. Participants were excluded if they had history of functional appliance therapy, cleft lip and/or palate and/or other craniofacial syndromes and fixed retention in the maxillary arch.

Data were extracted from clinical records and reference models obtained pre-treatment (*T*0), at the end of active treatment (*T*1) and 12 months post-treatment (*T*2). All orthodontic study model-based measurements were taken by two investigators using a TESA SHOP-Cal digital callipers (Resolution 0.01 mm).

The stability of the maxillary anterior teeth was assessed using two assessment tools: Little’s Irregularity Index and the Orthodontic Alignment Index (OAI). Little’s Index involves a cumulative score of contact point displacement involving five contact points in the inter-canine region. The OAI was developed and piloted to provide a more detailed assessment of minor orthodontic issues affecting maxillary labial segment alignment (“Appendix 1”).

A comparison of stability was made between subjects with a Class I incisor relationship (2–4 mm) and a residual overjet (> 4 mm) at least 12 months following the completion of fixed appliance-based treatment. Two investigators (DP and CD) were calibrated using both indices. Intra-examiner and inter-examiner reliability was assessed using the intra-class correlation coefficient (ICC).

All data were entered into Microsoft Excel™ for descriptive analysis and later transferred to the Statistical Software Stata 17™ (StataCorp, TX, USA). Descriptive statistics included mean values and standard deviations for continuous data. Participants were categorised dichotomously into normal (2–4 mm) and increased (in excess of 4 mm) overjet groups. Data were normally distributed; therefore, linear regression analysis was used to assess the effect of overjet at debond (*T*1) on the stability of maxillary anterior alignment 12 months post-treatment (*T*2). Statistical significance was set at *P* < 0.05.

### Development of the Orthodontic Alignment Index (OAI)

Twenty pre- and post-treatment models were obtained from local archives to evaluate the possible manifestations of instability in the maxillary anterior segment. Based on the previous research [3], a number of possible features of orthodontic instability including both horizontal and vertical change, reciprocal rotations and tip and torque changes have been shown to evoke negative responses from patients. Each occlusal feature was ranked in order of severity. A supplementary instability feature (spacing) was identified in the upper labial segment. These findings were used to develop a method of grading occlusal discrepancy according to six categories: horizontal discrepancy, spacing, vertical displacement, reciprocal rotations, tip and torque anomalies.

A panel of 10 qualified orthodontic clinicians was randomly selected to assess 10 sets of pre-treatment study models using the newly developed index. Feedback was used to refine the scoring system. The index was further piloted by a panel of 63 orthodontic clinicians recruited at the British Orthodontic Conference, in order to validate the index and determine inter-examiner reliability.

Participants were asked to score three maxillary pre-treatment plaster study models using the new index and to provide feedback on whether the scoring system fairly reflected aesthetic issues relating to the alignment of teeth. Free-text boxes for suggestions on how the system could be improved were also included.

Intra-class correlation coefficient (ICC) estimates and their 95% confidence intervals were calculated using StataCorp 15 TM (LLC Stata Statistical Software, 2017) using the pilot data. The intra-class correlation coefficient (ICC) was 0.72 for the 63 orthodontists. Hence, there was moderate-to-good inter-rater agreement [4], and further refinement of the index was undertaken.

Feedback from the pilot studies was used to refine the scoring system and assign weighting factors to each category according to severity to finalise the index (“Appendix 1”). The categories were refined as follows:

### Horizontal

An objective assessment of the most severe contact point displacement was made with a maximum score of 12 for more than one tooth with both contact points displaced more than 2 mm in the same direction.

### Spacing

An objective assessment of the presence or absence of spacing in the upper labial segment, with a maximum score of four points for spacing present in more than two areas.

### Vertical

Objective assessment of the most severe vertical discrepancy between pairs of incisors, i.e. upper central incisors, upper lateral incisors, and the adjacent lateral and central incisor (more than 1.5 mm in either direction), with a maximum score of eight points.

### Reciprocal rotations

Obvious rotations without contact point displacement, i.e. adjacent teeth rotated in the same direction without contact point displacement, with the score being given to the distance measured from the maximum point of rotation to the arch form (in mm), with a maximum score of two points.

### Tip

A subjective assessment of mesio-distal angulation on any tooth in the upper labial segment, with a maximum score of two points given for more than two teeth involved.

### Torque

Subjective perception of bucco-palatal orientation on any tooth in the upper labial segment, with a maximum score of two points given for more than two teeth involved.

## Results

### Reliability and validation of the Orthodontic Alignment Index

Following the pilot studies, the final refined index was tested again for inter-examiner reliability using five experienced clinicians on three new upper study models. ICC (two-way random, single measures, absolute agreement) was 0.99 (95% CI 0.98, 1.00) showing excellent inter-examiner agreement [4].

**Fig. 1:**
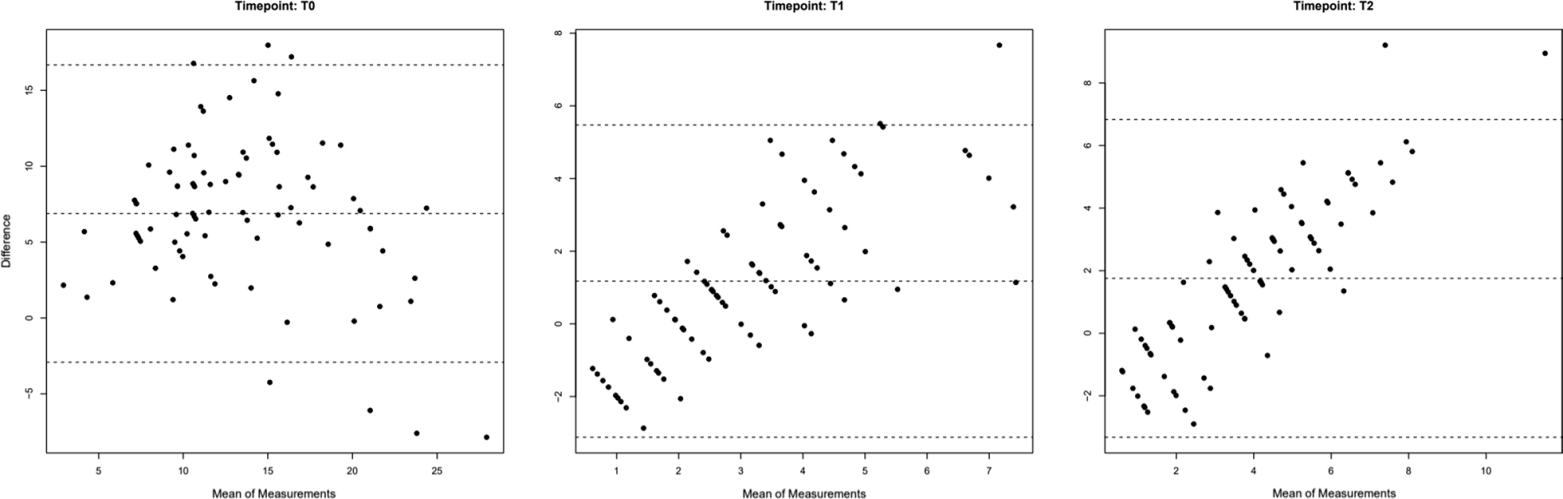
Bland–Altman plots to compare the Orthodontic Alignment Index and Little’s Irregularity Index at *T*0 (pre-treatment), *T*1 (debond) and *T*2 (12 months post-treatment)

The reliability of the two primary investigators was also assessed; the intra-examiner reliability ranged between 0.908 and 0.989 indicating moderate-to-excellent reliability and the inter-examiner reliability score for LII was 0.89 and for OAI was 0.98 also indicating moderate-to-excellent reliability [4]. The Bland–Altman method was used to measure the agreement between the two stability indices (Fig. 1). There was a positive association between both indices at debond (*T*1) and at the 12-month post-treatment review (*T*2), suggesting that agreement between the indices was good.

### Overall results

**Fig. 2:**
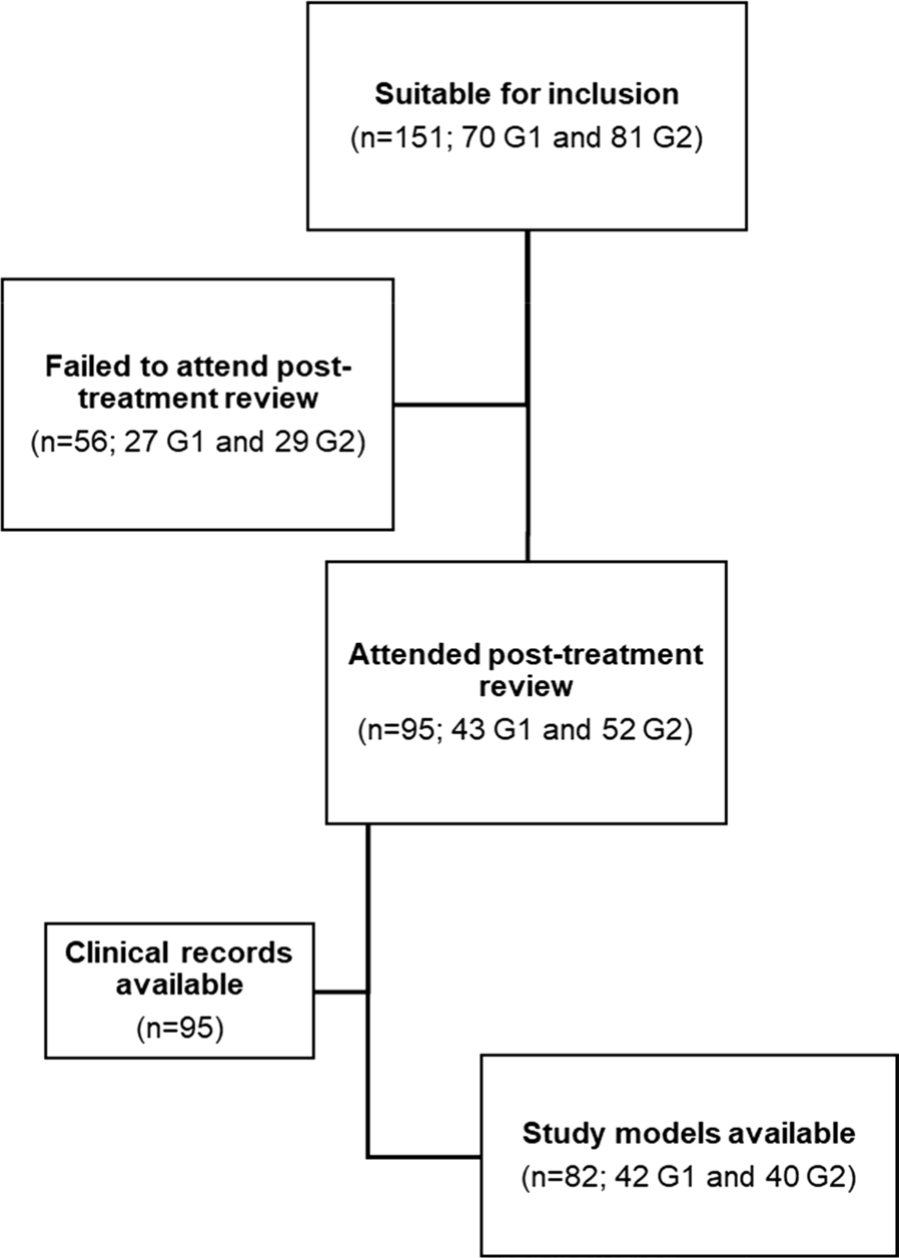
Participant flow and availability of records (Group 1: residual overjet group; Group 2: normal overjet group)

One hundred and fifty-one participants were suitable for inclusion; however, fifty-six participants failed to attend their post-treatment review. Of the remaining 95 participants, a complete set of records were not available for 13 participants (Fig. 2). Pre-treatment characteristics were reasonably well-matched between the groups with a mean age pre-treatment of 14.04 (SD 3.03) years (Table 1).

**Table 1:** Pre-treatment characteristics, treatment and retention regimes (Group 1: Residual overjet group; Group 2: Normal overjet group; PAE: Pre-adjusted edgewise appliance)

Variable	Group 1 (*n* = 42)		Group 2 (*n* = 40)	
Age pre-treatment mean (SD)	14.27 (2.93)		13.81 (3.15)	
	*n*	%	*n*	%
*Pre-treatment characteristics*				
Gender				
Male	18	43	19	48
Female	24	57	21	52
Incisor relationship				
Class I	10	24	11	28
Class II div 1	24	57	18	45
Class II div 2	5	12	5	12
Class III	3	7	6	15
*Treatment phase*				
Extraction-based treatment				
No	20	48	13	33
Yes	22	52	27	67
Maxillary retention				
VFR	37	88	38	95
Hawley	4	10	2	5
Other	1	2	0	0
None	0	0	0	0
Subjective compliance (12 months)				
Full-time	2	5	1	3
Every night	13	31	5	13
A few nights per week	20	48	26	64
No wear	6	14	8	20
Lip competency				
Yes	27	64	30	75
No	15	36	9	23
Not recorded	0	0	1	2

### Treatment and retention regimes

All participants were treated with pre-adjusted edgewise appliances with the majority treated on an extraction basis (*n* = 49, 60%) (Table 1). All participants received removable retention at the end of treatment with most subjects (*n* = 75, 91%) having a vacuum-formed retainer. Only 6 (7%) participants were given a Hawley retainer (Table 1).

At the post-treatment follow-up, the majority of participants reported wearing their retainers a few nights a week (*n* = 46, 56%). Subjective compliance was similar in both groups. Fourteen (17%) subjects reported not wearing their retainer, whilst 3 (4%) respondents reported full-time wear (Table 1).

### Post-treatment changes and stability

Overall, a deterioration in alignment when assessed using the OAI was observed with a mean increase of 1.07 (SD 2.86) points. An increase of 0.88 (SD 3.03) and 1.28 (SD 2.70) points was observed in the residual and normal overjet groups, respectively. A deterioration in LII in the maxillary anterior region was also seen in both groups with a mean increase of 0.49 mm (SD 1.11) from the end of treatment to the post-treatment review. A slightly greater increase was observed in the normal overjet group (0.52 mm, SD 1.04) than in the residual overjet group (0.47 mm, SD 1.19) (Table 2).

**Table 2:** Treatment-induced and post-treatment changes (Group 1: Residual overjet group; Group 2: Normal overjet group)

Study model-based measurements	Pre-treatment (*T*0)		Debond (*T*1)		Orthodontic correction (*T*1–*T*0)		12 months post-treatment (*T*2)		Post-treatment changes (*T*2–*T*1)				Post-treatment changes (*T*2–*T*1) as % of orthodontic correction	
	Group 1 (*n* = 42)	Group 2 (*n* = 40)	Group 1 (*n* = 42)	Group 2 (*n* = 40)	Group 1 (*n* = 42)	Group 2 (*n* = 40)	Group 1 (*n* = 42)	Group 2 (*n* = 40)	Group 1 (*n* = 42)		Group 2 (*n* = 40)		Group 1 (*n* = 42)	Group 2 (*n* = 40)
	Mean (SD)		Mean (SD)		Mean (SD)		Mean (SD)		Mean				Mean (%)	
									(SD)	%	(SD)	%		
OAI score	17.95 (5.31)	15.58 (4.98)	3.83 (2.76)	3.68 (2.43)	− 14.12 (5.73)	− 11.90 (5.73)	4.71 (3.59)	4.95 (3.04)	0.88 (3.03)	23	1.28 (2.70)	35	6	11
LII (mm)	10.84 (6.97)	8.94 (5.4)	2.67 (0.91)	2.49 (1.23)	− 8.17 (7.11)	− 6.45 (5.36)	3.13 (1.19)	3.01 (1.19)	0.47 (1.19)	17	0.52 (1.04)	21	6	8
Overjet (mm)	4.96 (3.20)	4.35 (2.54)	4.99 (0.84)	3.12 (0.56)	0.03 (3.24)	− 1.23 (2.64)	4.86 (1.09)	3.28 (0.89)	− 0.13 (0.89)	− 3	0.16 (0.76)	5	473	13

Clinically relevant instability was set as a score difference of greater than 1 point when using the OAI, and of more than 1 mm difference in irregularity using the LII over the study period. Based on these thresholds, using the OAI, 64% of participants in the residual overjet group and 72% of participants in the normal overjet group were considered to have unstable outcomes. Using LII, 31% of the residual and 32% of the normal overjet group were considered unstable (Table 3).

**Table 3:** Assessment of post-treatment stability

	Residual overjet group (*n* = 42)				Normal overjet group (*n* = 40)			
	*n*	%	*n*	%	*n*	%	*n*	%
OAI	Stable < 1		Unstable ≥ 1		Stable < 1		Unstable ≥ 1	
	15	36	27	64	11	28	29	72
LII	Stable < 1 mm		Unstable ≥ 1 mm		Stable < 1 mm		Unstable ≥ 1 mm	
	29	69	13	31	27	68	13	32

Linear regression modelling, with the OAI as a measure of instability, demonstrated a mean difference of 0.39 points more instability in the normal overjet group compared to the residual overjet group. This finding was not statistically significant (*P* = 0.538). After adjusting for baseline alignment levels using OAI and LII (*T*0), the mean difference between both overjet groups was 0.56 points (*P* = 0.389). Additional adjustments were made accounting for possible confounding factors including pre-treatment age, gender, extraction protocol, retention regime and compliance. However, no statistically significant associations were observed. Similar results were observed when LII was used as a measure instability (Table 4).

**Table 4:** Linear regression analysis of the effect of a residual overjet (*T*1) on post-treatment stability (*T*2) using the Orthodontic Alignment Index (OAI) and Little’s Irregularity Index (LII), adjusting for baseline alignment (*T*0) and confounding factors (Coef: coefficient)

Indices	Independent variable	Coef	*P*	95% Confidence interval	
				Lower	Upper
OAI	Overjet (*T*1)	0.56	0.389	− 0.70	1.86
	Age (*T*0)	0.57	0.388	− 0.74	1.87
	Gender	0.53	0.409	− 0.73	1.79
	Extraction (yes/no)	0.56	0.403	− 0.77	1.90
	Extraction pattern	0.54	0.439	− 0.84	1.92
	Retention regime	0.50	0.455	− 0.82	1.82
	Retention compliance	0.65	0.340	− 0.69	1.98
LII	Overjet (*T*1)	0.14	0.577	− 0.36	0.63
	Age (*T*0)	0.15	0.540	− 0.33	0.63
	Gender	0.13	0.592	− 0.35	0.61
	Extraction (yes/no)	0.12	0.625	− 0.38	0.62
	Extraction pattern	0.09	0.721	− 0.42	0.61
	Retention regime	0.18	0.477	− 0.32	0.67
	Retention compliance	0.06	0.824	− 0.44	0.55

## Discussion

At the 12-month review, a positive relationship was noted for LII and OAI; for every 1-mm increase in LII at *T*2, an increase of essentially 2 points of OAI at *T*2 was expected (*P* < 0.001). This suggests increased sensitivity of OAI in evaluating relapse in the upper anterior region. This distinction may be related to the insensitivity of LII to the evaluation of spacing, mutual rotations (without contact point displacement) and vertical discrepancies, which are prone to change following the completion of orthodontic treatment [5]. As such, the OAI may have potential as a means of evaluating post-treatment change more accurately.

Previous studies have confirmed poor reproducibility of contact point measurements between examiners in relation to LII scores with over 85% of individual contact point scores demonstrating a mean difference of > 20% between measurements [6]. Additionally, the LII assigns a cumulative score for contact point displacements; this may generate a high score for multiple evenly dispersed contact point displacements, which may be clinically irrelevant. Conversely, the OAI assesses the greatest single contact point displacement, therefore ensuring relapse is more sensitive to noticeable contact point displacements which are more clinically significant. Based on the previous research [3], other features of orthodontic instability including vertical change, reciprocal rotations and tip and torque changes may evoke negative responses from patients. Notwithstanding this, it would be useful to verify the utility of the OAI by relating the obtained scores to the perceptions of laypeople and patient cohorts, and on the basis of adequately powered prospective studies.

As patients having fixed retention were omitted from the analysis, the primary retention method provided was vacuum-formed retainers (91%). This is in keeping with international practice [7]. Only 17% reported no retainer wear at the 12-month review. The majority (83%), therefore, reported at least part-time wear of retainers at their 12-month review which compares favourably with other studies [8]. However, patient-reported compliance levels are typically overestimated [9] potentially confounding the estimates. The use of imbedded electronic monitoring sensors are a means of providing a more accurate impression of wear duration.

Post-treatment alignment of the maxillary anterior dentition was relatively unstable; OAI increased by a mean of 1.28 and 0.88 points in the normal and residual overjet groups, respectively. Similarly, Rowland et al*.* (2007) demonstrated a median increase in LII in the maxillary labial segment in participants who were prescribed a Hawley (0.51 mm) or a vacuum-formed (0.26 mm) retainer for up to 6 months following the completion of treatment [10]. Minor post-treatment changes were also observed in other retrospective studies evaluating long-term stability [11–13]. As such, the level of post-treatment change is in keeping with other studies and likely reflects suboptimal adherence with removable retainer wear. Sadowsky et al. demonstrated an increase in LII of 1.1 mm in participants from post-treatment to post-retention [14]. The increased relapse in irregularity experienced may be explained by the retrospective nature, the small sample size and the longer follow-up period (> 5 years) [14]. Whilst this study involved a shorter-term evaluation, a meaningful assessment of stability can be achieved at 12 months as a high proportion of relapse is known to occur soon after treatment ceases with the rate of change reducing over time [15]. Post-treatment irregularity changes were also significantly lower than in other long-term studies which examined relapse in the lower labial segment [16, 17]. However, these studies had a much longer follow-up than the above study; therefore, participants were more susceptible to age-related changes which contribute lower incisor irregularity [18].

The lack of observed difference between the normal and residual overjet groups may relate to the limited sample allied to the relatively short-term nature of the follow-up. Although there is a paucity of research on this topic, parallels may be drawn from studies which use occlusal indices to assess the influence of the quality of occlusion at debond. Nett and Huang (2005) demonstrated that overjet at debond based on the ABO-OGS index was not influential on the post-treatment occlusion at 10 years. However, no attempt was made to differentiate between participants with an increased or normal overjet at debond [19]. Contradictory results were observed by Ormiston et al. [20] in a retrospective analysis of ‘stable’ and ‘unstable’ occlusions. Participants who had post-treatment occlusions which were deemed unstable (PAR score change: > 10) had an increased overjet at debond compared to participants in the stable group. Although the difference was not statistically significant, it does suggest at least a potential influence of overjet on instability. However, the study was not powered to specifically evaluate the effect of overjet on instability and other confounding factors may have had a greater influence [20].

Of the 151 participants suitable for inclusion, 56 failed to attend their 12-month post-treatment review. The dropout rate was influenced by the local effects of the COVID-19 pandemic [21]. Despite this, a response rate of 63% was observed which compares favourably to previous retention studies [9]. A further potential limitation is the exclusion of subjects with maxillary fixed retention and the confounding effects associated with variable reporting of retainer wear. It is therefore conceivable that the level of post-treatment change observed may be higher than would have presented had fixed retention been used, and indeed that the effect of compliance may have diluted any potential impact of final overjet on stability of anterior alignment. Notwithstanding this, there is evidence that the benefit of fixed retention may not emerge until the medium term (up to 4 years post-treatment) as compliance with removable retainers begins to wane [22, 23]. Moreover, the avoidance of fixed retention is not likely to have a differential effect between the two treatment groups. It is important to highlight, however, that maturational changes continue to influence dental alignment over protracted periods. It is therefore conceivable that the effect of increased overjet may become apparent over a longer period of evaluation. Further research using our bespoke index would be required to evaluate this contention.

## Conclusions

The novel Orthodontic Alignment Index may be a useful tool in the assessment of post-treatment stability. Limited post-treatment change in alignment of the maxillary anterior dentition was observed in both groups. The degree of relapse post-treatment was independent of the magnitude of overjet at the end of treatment. On the basis of this retrospective evaluation, the attainment of a normal overjet at the end of treatment may not influence the post-treatment stability of the maxillary anterior dentition up to 12 months post-treatment.

## Data Availability

The datasets used and/or analysed during the current study are available from the corresponding author on reasonable request.

## Abbreviations

ABO-OGS: American Board of Orthodontics Objective Grading System
CD: Ciarán Devine
Coef: Coefficient
CSF: Circumferential supracrestal fiberotomy
DP: Devaki Patel
G1: Group 1 (residual overjet group)
G2: Group 2 (normal overjet group)
ICC: Intra-class correlation coefficients
LII: Little’s Irregularity Index
mm: Millimetre
*n*: Sample size
NHS: National Health Service
OAI: Orthodontic Alignment Index
*P*: Probability value
PAE: Pre-adjusted edgewise appliance
PAR: Peer assessment rating
SD: Standard deviation
*T*0: Pre-treatment (baseline)
*T*1: Debond
*T*2: One year post-treatment
U.K.: United Kingdom
VFR: Vacuum-formed retainer
